# Readily accessible CT scoring method to quantify fibrosis in IPF

**DOI:** 10.1136/bmjresp-2020-000584

**Published:** 2020-06-10

**Authors:** Emily Fraser, Victoria St Noble, Rachel K Hoyles, Rachel Benamore, Ling-Pei Ho

**Affiliations:** 1 Oxford Interstitial Lung Disease Service, Oxford University Hospitals NHS Foundation Trust, Oxford, Oxfordshire, UK; 2 Department of Thoracic Imaging, Oxford University Hospitals NHS Foundation Trust, Oxford, Oxfordshire, UK; 3 MRC Human Immunology Unit, Weatherall Institite of Molecular Medicine, Oxford, UK

**Keywords:** imaging/CT MRI etc

## Abstract

**Introduction:**

There is currently no readily accessible measure to specifically quantify the amount of fibrosis in idiopathic pulmonary fibrosis (IPF). Such a measure could isolate contribution of fibrosis from other comorbidities to lung function abnormality and deterioration of disease, and potentially help determine if there has been response to antifibrotic treatment.

**Methods:**

In a pilot study of 39 IPF patients, we used a CT-based visual scoring method to examine the correlation between the sum of all fibrotic features (all traction bronchiectasis, ground glass with traction bronchiectasis, honeycombing and reticulation; referred to as Total Fibrosis Score, TFS) or the individual fibrotic features, with lung function, Composite Physiologic Index (CPI) and time to death in the 5 years following CT measurement.

**Results:**

TFS measurements were highly reproducible (r=0.982; p<0.001) and correlated significantly with TLCO, FVC and CPI. Traction bronchiectasis score was superior to others in its correlation to lung function and CPI, and as good as TFS. TFS and traction bronchiectasis score were also the best correlates (individually) to time to death (r=0.60 for both, and p=0.002 and p=0.004, respectively).

**Conclusion:**

We suggest that TFS and our 6-slices method of quantifying traction bronchiectasis on CT scans could be readily accessible and simple methods of quantifying lung fibrosis in IPF. These scores could assist in determining if clinical deterioration is due to worsening fibrosis, for correlation of research findings to amount of lung fibrosis, and to stratify patients for established drug treatment and clinical trials. Our findings also provide a basis for larger studies to validate these findings and determine if the scores could measure change in fibrosis.

**Key messages**

**What is the key question?**

- Can a visual method of assessing the amount of Usual Interstitial Pneumonia (UIP) pattern fibrosis on thoracic CT scan provide a method to specifically quantify the amount of fibrosis in idiopathic pulmonary fibrosis (IPF).

**What is the bottom line?**

- A CT-based visual scoring method comprising the sum of UIP fibrotic features or just the amount of traction bronchiectasis could be used to quantify lung fibrosis in IPF.

**Why read on?**

- The paper describes the methods and evidence to support this.

## Introduction

Idiopathic pulmonary fibrosis (IPF) is a chronic fibroproliferative disease with a median survival of 3–5 years from diagnosis.^1^ Establishing the extent of fibrosis at baseline and at follow-up can have prognostic and therapeutic implications but there are limited methods for doing this. Worsening breathlessness can signify advancement of disease, but is subjective and complicated by other comorbidities. Physiological parameters like the forced vital capacity (FVC) and transfer factor for carbon monoxide (TLCO) do not always reflect the extent of fibrosis and can be unreliable in the context of coexistent disease, such as emphysema, pulmonary hypertension and cardiac failure.^2^ The Composite Physiologic Index (CPI), a formula that incorporates forced expiratory volume in 1 s (FEV1), FVC and TLCO to overcome the confounding influence of airways disease^3^ accommodates the presence of emphysema but does not specifically measure fibrosis. In practice, issues such as poor patient technique can also result in readings that overestimate disease severity. A further limitation in using lung function as a measure of disease severity is that the range of ‘normal’ values lies between 80% and 120%, which may misrepresent presence of fibrosis in relatively healthy individuals.^4^ Lung function may also not be sensitive enough to detect accumulation of fibrosis—Oda *et al*^5^ observed a subgroup of patients with progressive fibrotic change on CT over 6 months which was not associated with a fall in FVC. In the era of increasing antifibrotic drugs (pirfenidone and nintedanib) that specifically slows down the progression of fibrosis, a method that measures the impact of these drugs on the accumulation of fibrosis could refine the use of these drugs. In addition, such a measure could complement lung function for selection and stratification of patients for established and new antifibrotic drugs.

High-resolution CT (HRCT) scans offer the possibility of measuring disease severity more accurately and sensitively, by focusing on fibrotic changes.^6^ Several computer-generated and visual approaches have been reported^5 7–19^ to provide a measure of disease severity in IPF. Software that can detect textural differences within the lung corresponding to specific CT patterns of fibrosis (eg, CALIPER) are promising particularly for variables not quantifiable by visual CT methods, for example, quantification of vessel in prognostication^17^ but have not been widely adopted despite several large studies,^18 19^ in part due to the costs and further training involved. Thus, a method that uses currently available technology and terminology,^20^ which is readily accessible, continues to have a role, particularly clinically. We set out to design a simple, short and readily accessible CT scoring system that quantifies fibrosis with the aim of identifying a scoring method that could contribute to research studies, patient selection and stratification in clinical trials for IPF, and determining the contribution of fibrosis to clinical deterioration.

## Methods

### Patients

Thirty-nine patients were recruited from the Oxford Interstitial Lung Disease service, who had a multidisciplinary team-defined diagnosis of IPF, using criteria from the 2011 American Thoracic Society/European Respiratory Society (ATS/ERS)statement^1^ as patients were recruited from 2013 to 2015 (demographic details in table 1). Patients fitting these criteria were recruited during clinical attachment months for the first author in this period. A pragmatic target of 50 was set for a pilot study. Forty-nine per cent had a ‘definite’ diagnosis and 51% a ‘probable’ UIP on CT scan. The same proportions were observed when the 2018 criteria^21^ was used to classify CT changes for UIP.

**Table 1:** Demographic data for patient cohort

N	39
Age mean (range)	72.5 (57–85)
Gender	
Male	34 (87%)
Female	5 (13%)
IPF diagnosis	
Definite	19 (49%)
Probable	20 (51%)
Smoking status	
Ex-smoker	29 (74%)
Never-smoker	10 (26%)
Antifibrotic therapy	
Yes	8 (20%)
No	31 (80%)
FVC; mean (IQR;SD)	70.9% (63.1–80.7;14.1)
TLCO; mean (IQR;SD)	49.5%(35.0–57.8;14.9)
CPI; mean (IQR;SD)	47.8% (41.2–57.6;12.3)
Median period of follow-up in months, mean (IQR;SD)	29.6 (9.1–30.2;14.3)
No (%) alive at follow-up	13 (33)

Pulmonary function test refers to % predicted for age, gender and height. unless stated, % in parentheses refer to % of cohort.

CPI, Composite Physiologic Index; FVC, forced vital capacity; IPF, idiopathic pulmonary fibrosis; TLCO, transfer factor for carbon monoxide.

Patients with active cancer and coexistent lung disease (except for emphysema if the proportion was less than interstitial disease changes in the thoracic CT), were excluded. HRCT scans were performed for clinical reasons, and scored independently by two chest radiologists (RB and VSN, with 11 and 6 years experience in HRCT interpretation at the time of study), blinded to the clinical data and to each other’s analysis.

Lung function measurements, encompassing FVC, TLCO and FEV1, were collected from each patient within 6 months of CT imaging, with the exception of one case where the patient was unable to perform the lung function technique. The TLCO and FVC are quoted as a percentage of predicted normal values for age, sex and height. The CPI was calculated using the following formula: CPI=91 – (0.65 x % predicted TLCO) - (0.53 x %FVC) + (0.34 x % predicted FEV1) according to reference.^3^ Survival data were collected in the following 5 years from CT scanning. All-cause mortality was used in this study.

### CT protocol

CT scans were acquired using a 64-detector row CT scanner (LightSpeed VCT XT; GE Medical Systems, Milwaukee, Wisconsin, USA). Images were reconstructed using a high spatial resolution algorithm. A volumetric scan was performed with 0.625 mm slice thickness at an interval of 0.625 mm.

### Score design

**Figure 1:**
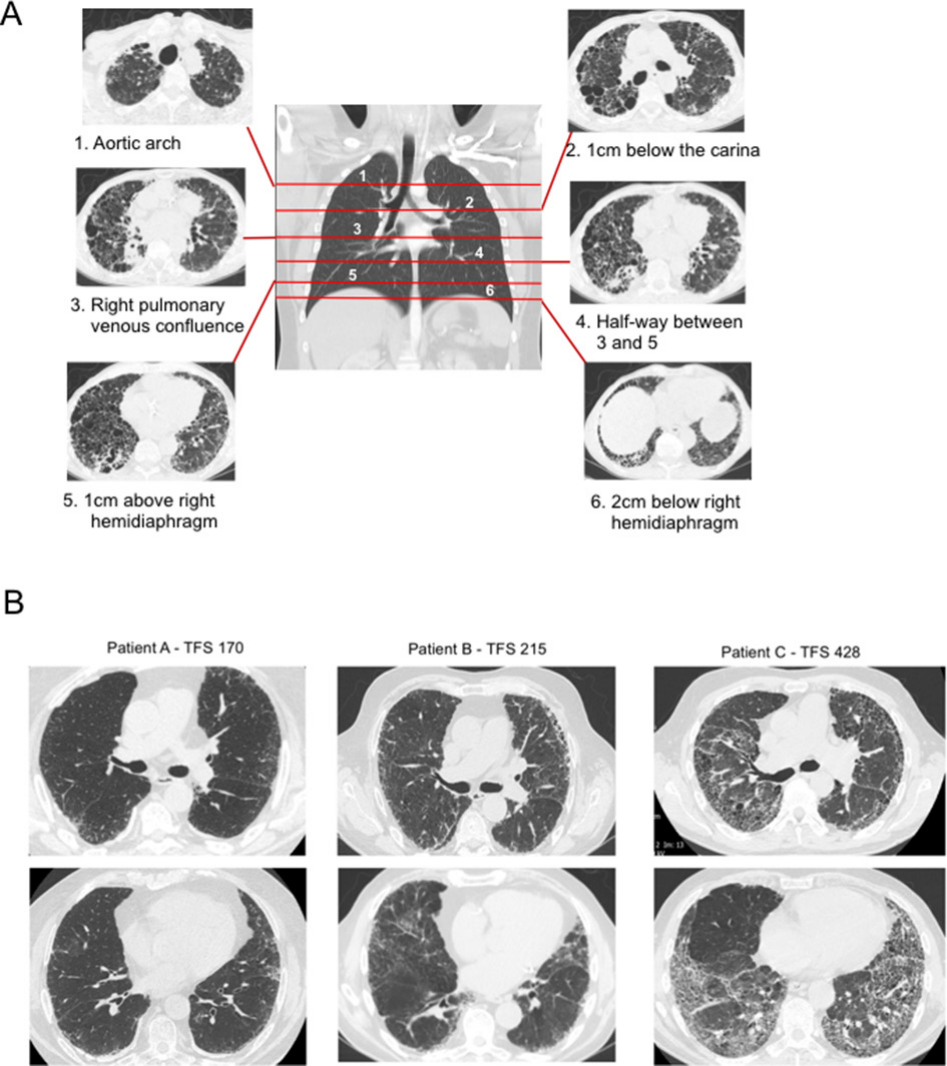
(A) Positions of the 6 slices of CT scan images (right and left) used for analysis of fibrotic features. (B) Examples of numerical scores of the TFS for three patients. TFS, Total Fibrosis Score.

We reviewed eight publications on visual scoring methods^7–14^ and adopted a modified method incorporating continuous variables for fibrotic features pertinent to IPF. Six anatomically defined axial sections of the thoracic HRCT were selected for analysis, using landmarks used by Edey *et al* (figure 1A).^7^ The first section was defined by the aortic arch; the second section was sited 1 cm below the carina; the third section was delineated by the pulmonary venous confluence and the fourth lay equidistant between the third and the fifth section. The fifth section was located 2 cm above the right hemidiaphragm and the sixth section was 1 cm below the right hemidiaphragm. These sections incorporated the upper, middle and lower lung zones but were weighted towards the lower zones due to the predilection of the disease to affect the lungs more basally (figure 1A). The lung sections selected for analysis by the first radiologist were then used by the second radiologist to enable direct comparison of the scores.

The proportion of honeycombing (‘H’), reticulation (‘R’), traction bronchiectasis (‘TB’) and ground glass opacification with TB (‘GGO+TB’; taken to signify fine fibrosis) within each section (right and left) was scored to the nearest 5%. ‘TB’ included that found within and outwith the ground glass changes. Ground glass changes without TB were not included in this score. Proportion of TB was calculated by estimating the percentage of lung which contained dilated bronchi on each representative CT section. The Total Fibrosis Score (TFS) was the sum of the scores of each fibrotic component (H, R, TB and ground glass with TB). Exemplars of different TFS values are shown in figure 1B.

All CT abnormalities were defined using standard Fleischner-based terminology.^20^ Amount of emphysema compared with interstitial lung disease were checked during scoring to ensure the former was less than the latter.

### Scores validation

TFS and the individual fibrosis scores were tested against % predicted FVC, % predicted TLCO and CPI measured within 6 months of the CT scan and time to death from CT scan.

### Statistical analysis

Interobserver reproducibility of the individual H, TB, TB +GGO and R scores was assessed using the Pearson correlation test. A Bland-Altman analysis was also performed to determine whether systematic deviation and bias existed between the paired measurements. The mean of the scores from the two radiologists were then used as the representative score for the correlation analyses. TFS and individual fibrotic components were examined for correlation with survival and lung function indices (TLCO, FVC and CPI) using Pearson correlation test. Statistical analysis was performed using GraphPad Prism (V.7).

## Results

**Figure 2:**
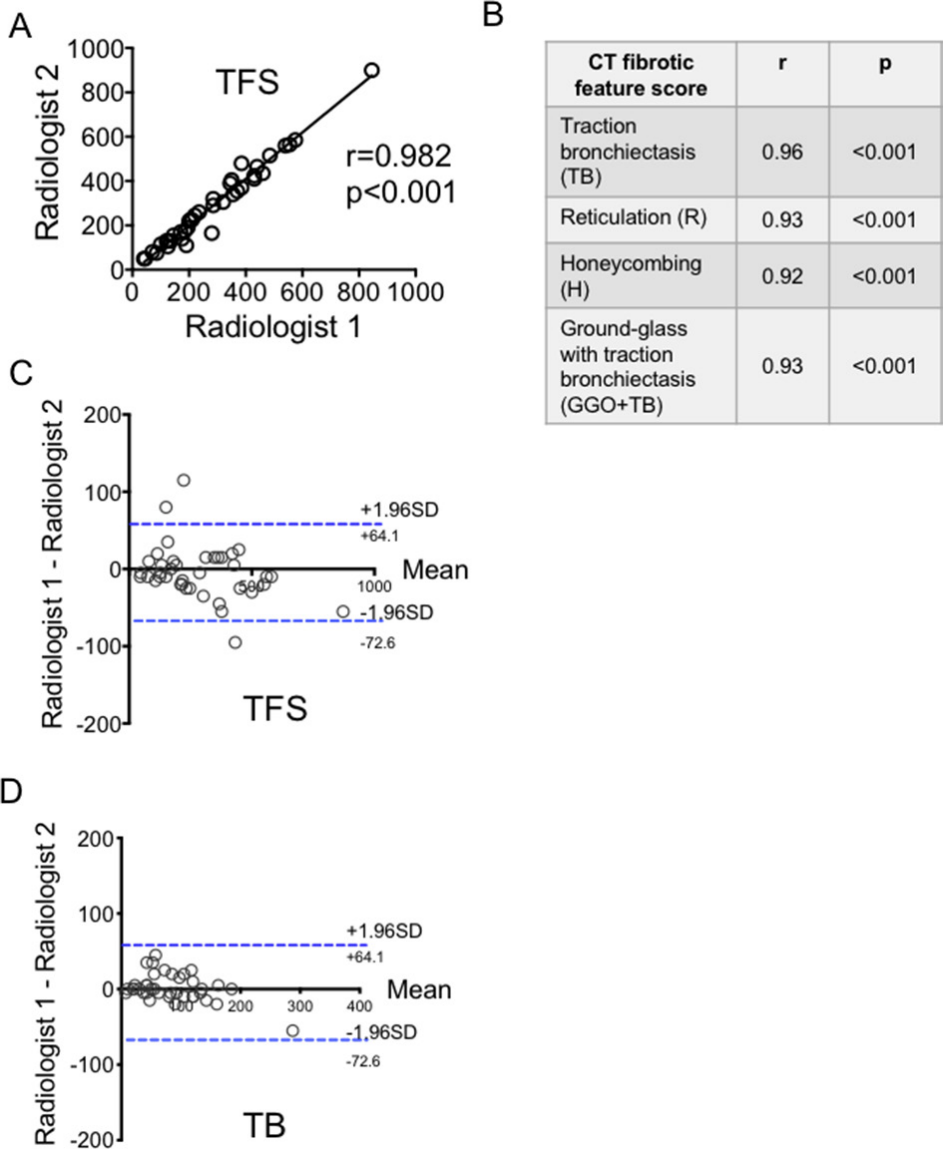
(A–D) Reproducibility of Total Fibrosis Score (TFS) and individual fibrotic components between two radiologists; and agreement analysis for TFS and TB. Analysis by Pearson correlation test and test for systematic bias and agreement were done using Bland-Altman plot.

TFS and the individual fibrotic component scores (H, R, TB and GGO+TB) showed excellent interobserver reproducibility. Comparing scores from the two radiologists, highly significant positive correlations were observed (r>0.90 and p<0.001, Pearson correlation test, figure 2A, B). Bland-Altman analysis of TFS and all individual CT fibrotic feature scores between the radiologists confirmed an absence of systematic deviation (TFS and TB shown in figure 2C, D).

**Figure 3:**
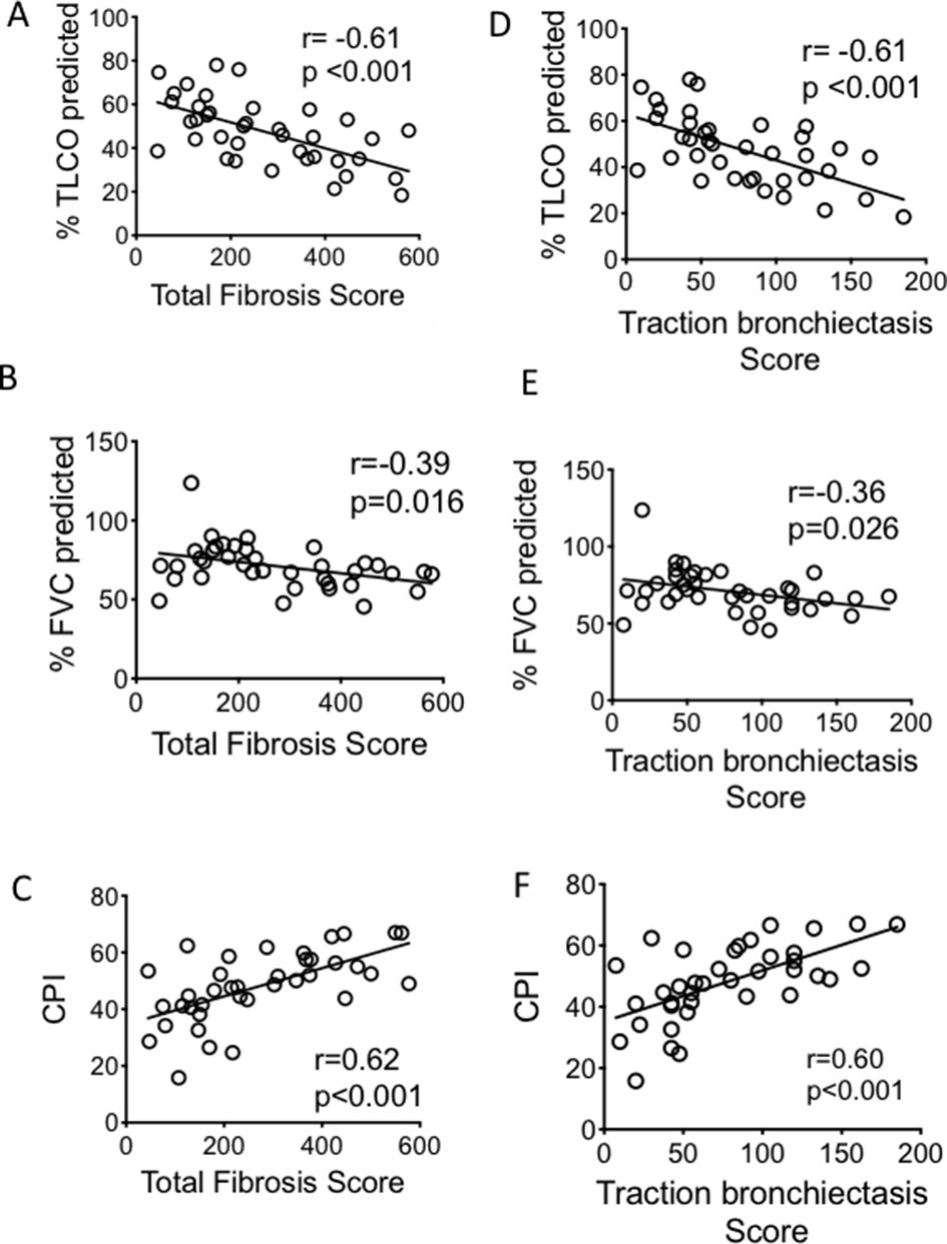
Correlation between the TFS (A–C) and traction bronchiectasis score (D–F) and lung function parameters and CPI. CIs for the correlation coefficients are as follows: −0.78 to −0.36; −0.63 to −0.08; 0.37 to 0.78; −0.78 to −0.37; −0.61 to −0.04; 0.38 to 0.78 for (A–F) respectively. All fitted lines are from least square regression analysis. Analysis of correlation performed with Pearson correlation test. CPI, Composite Physiologic Index; FVC, forced vital capacity; TLCO, transfer factor for carbon monoxide.

**Figure 4:**
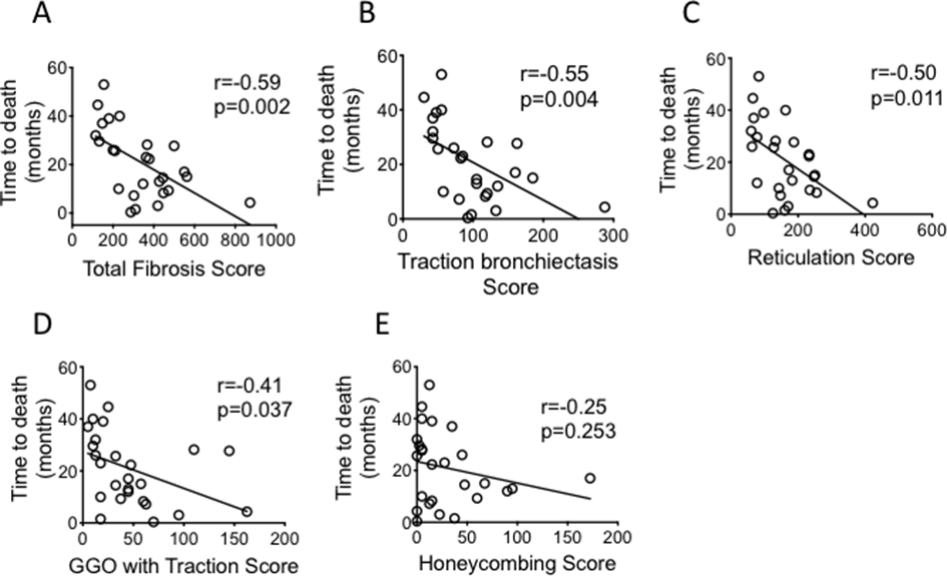
Correlations of Total Fibrosis Score and other individual fibrotic scores with time to death (as measured from the point of CT imaging). CIs for the correlation coefficients are as follows: −0.79 to −0.26; −0.77 to −0.20; −0.76 to −0.17; −0.69 to −0.03; −0.57 to 0.17 for (A–E) respectively. all fitted lines are from least square regression analysis. Analysis of correlation performed with Pearson correlation test. GGO, ground glass opacification.

TFS correlated significantly with TLCO, CPI and FVC (r=−0.610, p<0.001, r=0.619, p<0.001 and r=−0.390, p=0.016 respectively, figure 3A–C). Comparing correlation between the individual CT fibrotic features with lung function and CPI, TB showed the best correlation with all lung function parameters (table 2 and figure 3D–F). We followed the patient for 5 years to determine their mortality rate. Of the 39 patients enrolled, 26 died in this time interval. There was an inverse correlation between the TFS and survival (figure 4A) (r=−0.587, p=0.002). Of the individual CT fibrotic feature scores, TB was found to correlate most closely with time to death (r=−0.548, p=0.004, figure 4B–E), and was near identical to TFS. H extent was not significantly correlated with prognosis (figure 4E).

**Table 2:** Correlations of individual fibrotic components scores with lung function and CPI

CT fibrotic feature scores	TLCO R	TLCO, p value	FVC R	FVC, p value	CPI R	CPI, p value
Traction bronchiectasis	−0.61 (−0.78 to −0.37)	<0.001	−0.36 (−0.61 to −0.04)	0.026	0.60 (0.38 to 0.78)	<0.001
Reticulation	−0.49 (−0.71 to −0.21)	0.002	−0.32 (−0.58 to 0.00)	0.05	0.40 (0.10 to 0.64)	0.013
Honeycombing	−0.52 (−0.72 to −0.24)	0.001	−0.19 (−0.48 to 0.14)	0.260	0.45 (0.15 to 0.67)	<0.010
Ground glass with traction bronchiectasis	−0.31 (−0.57 to −0.01)	0.060	−0.34 (−0.60 to −0.02)	0.040	0.41 (0.10 to 0.64)	0.011

CIs to correlation coefficient in parentheses.

CPI, Composite Physiologic Index; FVC, forced vital capacity; R, reticulation; TLCO, transfer factor for carbon monoxide.

## Discussion

Our study describes a simple method of quantifying lung fibrosis in IPF patient, using readily available HRCT images that are accessible to all thoracic radiologists. The method used Fleischner radiology terms and standard CT acquisition methods, and takes 10–15 min for a total score (TFS) or 5 min for the abbreviated TB score. It is highly reproducible between our radiologists, and correlated with lung function and time to death, supporting its validity as a measure of the amount of fibrosis.

The value of these scores is in their ability to provide a direct measure of the amount of fibrosis in IPF, which can be used in research studies to test links of findings to fibrosis and in patient selection and stratification in clinical trials for IPF. Clinically, as it measures fibrosis specifically (in contrast to lung function which is affected by technique, cardiac status, pulmonary hypertension), the TFS or TB scores could help differentiate fibrotic progression from other causes in periods of clinical deterioration. The method is not intended to replace lung function measurements, rather to complement it.

Our method was adapted from several previous visual methods^7–14^ but to our knowledge is the only one using 6 slices, with all continuous variables (ie, TB measured in the same way as all other CT features unlike Edey and Walsh’s scores).^7 10^ In addition, ground glass without presence of admixed TB was excluded from the fibrosis score; with the intention of making this a fibrosis-specific continuous score, for IPF.

In our score, measuring total TB (as a sum of the % per section of the six defined slices) showed similar correlation to prognosis as measuring the sum of all the individual fibrotic features. This has support from Edey and Walsh’s larger studies^7 10^ where severity of TB was also strongly associated with survival. In our study, correlation with CPI, TLCO, FVC and time to death were examined to validate TFS and TB scores as a measure of lung fibrosis, rather than predictive measures.

In proposing this method, we acknowledge that automated methods may be the way forward for assessing the amount of fibrosis in IPF. However, there is currently no agreed automated CT measure, and this may still be out of reach of many radiology departments. Our method, particularly with the TB measurement, is quick and is readily accessible to all thoracic radiologists.

The study is also limited by the number of patients and lack of follow-up CTs to determine the sensitivity of the measure to capture change. Larger, more detailed studies, powered to test the correlation of its rate of change with treatment with antifibrotics, and its sensitivity to do so compared with other quantitative methods like CALIPER will be required to further evaluate its utility. An assessment of its ease and reproducibility in a broader range of radiologists will also be necessary. Currently, its greatest use is in measuring the amount of fibrosis at baseline, which could contribute to prognostication, research study analyses and determining if worsening of symptoms is due to progression in fibrosis. For the last, TFS would be a better measure than TB as it would provide greater likelihood of capturing all types of change.

In summary, the TFS and TB methods offer a readily accessible method of measuring the amount of fibrosis in IPF patients. They show high reproducibility, are validated against lung function and CPI and correlate with prognosis. Larger studies could confirm its utility as a specific measure of fibrosis and its sensitivity in detecting change after antifibrotic treatment.
